# Effects of Motivation and Medication on Electrophysiological Markers of Response Inhibition in Children with Attention-Deficit/Hyperactivity Disorder

**DOI:** 10.1016/j.biopsych.2009.09.029

**Published:** 2010-04-01

**Authors:** Madeleine J. Groom, Gaia Scerif, Peter F. Liddle, Martin J. Batty, Elizabeth B. Liddle, Katherine L. Roberts, John D. Cahill, Mario Liotti, Chris Hollis

**Affiliations:** a Developmental Psychiatry, University of Nottingham, Nottingham, United Kingdom; b Department of Experimental Psychology, University of Oxford, Oxford, United Kingdom; c Behavioural Sciences, Division of Psychiatry, University of Nottingham, Nottingham, United Kingdom; d Department of Psychology, Simon Fraser University, Burnaby, British Columbia, Canada

**Keywords:** ADHD, electrophysiology, motivation, response inhibition, stimulant medication

## Abstract

**Background:**

Theories of attention-deficit/hyperactivity disorder (ADHD) posit either executive deficits and/or alterations in motivational style and reward processing as core to the disorder. Effects of motivational incentives on electrophysiological correlates of inhibitory control and relationships between motivation and stimulant medication have not been explicitly tested.

**Methods:**

Children (9–15 years) with combined-type ADHD (*n* = 28) and matched typically developing children (CTRL) (*n* = 28) performed a go/no-go task. Electroencephalogram data were recorded. Amplitude of two event-related potentials, the N2 and P3 (markers of response conflict and attention), were measured. The ADHD children were all stimulant responders tested on and off their usual dose of methylphenidate; CTRLs were never medicated. All children performed the task under three motivational conditions: reward; response cost; and baseline, in which points awarded/deducted for inhibitory performance varied.

**Results:**

There were effects of diagnosis (CTRL > ADHD unmedicated), medication (on > off), and motivation (reward and/or response cost > baseline) on N2 and P3 amplitude, although the N2 diagnosis effect did not reach statistical significance (*p* = .1). Interactions between motivation and diagnosis/medication were nonsignificant (*p* > .1).

**Conclusions:**

Motivational incentives increased amplitudes of electrophysiological correlates of response conflict and attention in children with ADHD, towards the baseline (low motivation) amplitudes of control subjects. These results suggest that, on these measures, motivational incentives have similar effects in children with ADHD as typically developing CTRLs and have additive effects with stimulant medication, enhancing stimulus salience and allocation of attentional resources during response inhibition.

Attention-deficit/hyperactivity disorder (ADHD) is a neurodevelopmental disorder characterized by age-inappropriate levels of hyperactivity, impulsivity, and inattention. Children with ADHD exhibit a range of cognitive difficulties, including impaired inhibition of the prepotent motor response in go/no-go and stop signal tasks (1,2), leading some to suggest a central role for response inhibition deficits (3). Impaired response inhibition in ADHD is associated with underactivation of a fronto-striatal-thalamic circuit involved in cognitive control (4) and reduced amplitude of two event-related potentials (ERPs), the N2 and P3 (5–8), which in healthy individuals increase on trials requiring motor inhibition or conflict resolution (9,10).

Children with ADHD also show atypical regulation of motivational state: they respond to rewards when they are delivered immediately and with regularity but are less sensitive to rewards that are delayed or intermittent (11,12). It has been suggested that deficits in higher cognitive functions in ADHD, including response inhibition, conflict processing, and attention, are a consequence of impairments in the self-regulation of arousal and motivational state (13,14). This raises the interesting question of whether incentives given to enhance motivation will improve inhibitory performance in children with ADHD. With the exception of two studies (15,16), most behavioral studies examining this hypothesis have argued in favor of independence of inhibitory and motivational deficits (17–24). However, methodological weaknesses limit their interpretation, including the absence of a baseline (no or low motivation) (17,20); the presentation of baseline and/or motivated conditions in a fixed order such that, even after counterbalancing, order effects were uncontrolled and not always equivalent between groups (19,21,22); and baseline and motivational conditions delivered in separate sessions or hours apart, potentially weakening the context provided by the baseline (23). Another important question, which to our knowledge has not yet been explored, is whether there are effects of motivation on covert measures of neural function.

We investigated effects of motivation on neural correlates of response conflict and attention in ADHD using a go/no-go paradigm, in which two motivational conditions and a baseline (low motivation) condition were presented repeatedly, randomized within session. Electroencephalography (EEG) data were collected while children performed the task; amplitudes of N2 and P3 were measured. We predicted significantly smaller N2 and P3 amplitudes in the ADHD group than the control group. Incentives were delivered continuously and immediately. We therefore predicted that if inhibitory deficits are related to impaired motivation in ADHD, incentives would significantly enhance amplitudes of the ADHD group to at least the same level as the baseline (low motivation) amplitudes of the control group.

In addition, we investigated relationships between stimulant medication (methylphenidate [MPH]) and motivation in the ADHD group using a within-subjects design. Methylphenidate is believed to increase availability of dopamine in the striatum, enhancing salience of task-relevant stimuli (25,26), improving inhibitory performance (27–29), and normalizing latency (30) and amplitude (31) of N2 and P3. It is currently unknown whether other means of altering arousal, such as providing motivational incentives, have similar effects on neurocognitive function as those produced by stimulant medications, and there is relatively little research investigating the effects of medication on such correlates. If motivational incentives and stimulant medication produce complementary effects on neural correlates of response inhibition, this would provide some support for the combined use of medication and behavioral reinforcement in the treatment of ADHD. Although this approach was supported by initial findings of the Multimodal Treatment Study of Children with ADHD (MTA) (32) with lower medication doses required for symptom control in children receiving combined medication and behavioral management, there have, as yet, been no explicit, published comparisons of the effects of motivational incentives and methylphenidate on neural correlates of inhibitory control. If medication and motivation act synergistically, N2 and P3 amplitudes will be differentially larger in children with ADHD in the high-motivation conditions when medicated than when unmedicated. Alternatively, each intervention may result in independent improvements in neural functioning.

## Methods and Materials

Participants were 28 children with ADHD (27 male participants), aged 9 to 15 years, and 28 typically developing control subjects matched on age, gender, and socioeconomic status (SES). Ethical approval was granted by the Multicentre Research Ethics Committee (MREC). Informed written parental consent and verbal participant assent were obtained from all participants. Table 1 summarizes demographic and clinical information.

### Recruitment of ADHD Participants

**Figure 1:**
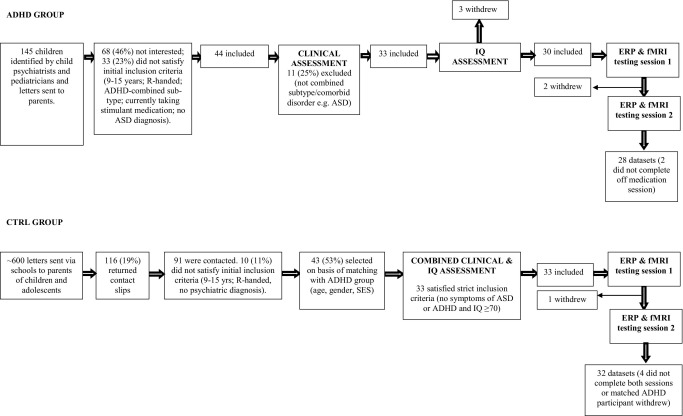
Flow chart showing recruitment and assessment of participants in control (CTRL) and attention-deficit/hyperactivity disorder (ADHD) groups. ASD, Autism Spectrum Disorder; ERP, event-related potential; fMRI, functional magnetic resonance imaging.

Figure 1 presents recruitment information. Children with a diagnosis of ADHD were referred to the study by child psychiatrists and pediatricians. An initial telephone interview screened for broad inclusion and exclusion criteria, before an in-depth assessment in person using the following measures: Strengths and Difficulties Questionnaire (SDQ) (33); Conners Rating Scale-Revised (Long Form) (34); Development and Well Being Assessment (DAWBA), a semistructured clinical interview (35); and Social Communication Questionnaire (SCQ) (36). With the exception of the SCQ, which was administered to parents only, all were completed by parents and teachers. This, along with the child's medical notes, formed the basis for a consensus diagnostic conference involving C.H. and another experienced child and adolescent psychiatrist. Only cases with a confirmed diagnosis of ADHD-combined subtype and an established clinical response to MPH were included, unless they met one of the following exclusion criteria: comorbid diagnosis (or symptoms above a specified threshold) of tic disorder, pervasive developmental disorder, learning disability (IQ <70) assessed using the Wechsler Abbreviated Scale of Intelligence (37), and neurological disorder. Children with comorbid anxiety disorder, oppositional defiant disorder (ODD) (*n* = 13, 46%), and conduct disorder (*n* = 8, 29%) were not excluded.

### Recruitment of Control Participants

Letters detailing the study were sent via local schools to parents. Right-handed children with no psychiatric diagnosis were selected for further assessment if they matched demographically a member of the ADHD group. Exclusion criteria were those listed above for the ADHD group in addition to score >5 on the hyperactivity subscale of the SDQ and/or score >1 SD above normative means on the Conners Rating Scale-Revised (Long Form) ADHD index. Anxiety, depression, and conduct problems were not excluded, although no control subjects met criteria for these disorders.

### Paradigm

The paradigm was a modified version of the visual go/no-go task, programmed using E-Prime (version 1.1, Psychology Software Tools). Stimuli were presented centrally on a color monitor positioned approximately 57 cm in front of participants. Interstimulus interval (ISI) was randomly jittered between 2.8 and 3.8 sec; stimulus duration was 100 msec. Participants were instructed to fixate on a central point and press a response button (Cedrus Superlab button box, Cedrus Corporation, San Pedro, California) each time a frequent “go” stimulus appeared and to refrain from responding to an infrequent “no-go” stimulus. There were 600 trials in total with 150 (25%) no-go trials, presented in three different motivational conditions (200 trials per condition). The paradigm was presented in the context of a “space” theme; go stimuli were green aliens measuring 43-mm height by 40-mm width; no-go stimuli were black aliens of equal size. Participants were encouraged to “catch” as many green aliens as possible while avoiding catching the black aliens. A time limit (reaction time [RT] cap) was imposed on go trials: participants lost 1 point for each slow or missed response (visual feedback given 1000 msec poststimulus) and gained 1 point for each timely response.

To minimize between- and within-subject variance in inhibition rate, the RT cap was dynamically altered throughout the experiment by a tracking algorithm. Prior to the start of the experiment, participants performed a practice session comprising 20 go trials, which used a staircase procedure to identify the shortest time within which they could respond. This value became the lower bound of the tracking algorithm during the main experiment (floor RT). The initial value of the tracking algorithm for each motivational condition was set at this value plus 200 msec. This RT cap was altered dynamically, based on performance on no-go trials: following failed inhibits (FI), the cap increased by 25 msec to improve the chance of success on the next no-go trial; each successful inhibit (SI) resulted in a 25 msec decrease. There was an upper bound of 900 msec for all participants.

### Motivational Conditions

Participants performed the go/no-go task in three different motivational conditions: baseline (low motivation); reward; and response cost. In the baseline condition, participants gained or lost 1 point following each SI or FI, respectively. In the reward condition, 5 points were given for each SI; in the response cost condition, 5 points were deducted for each FI. Thus, in the baseline condition, the value attached to inhibiting on a no-go trial was equal to that of responding quickly on a go trial. In the motivated conditions, the value attached to successfully inhibiting on a no-go trial was greater than the value attached to responding quickly on a go trial, resulting in relatively greater incentive to inhibit.

Stimuli were presented in five blocks, with each motivational condition randomly presented once within each block. At the end of each condition, a visual display detailed how many points had been lost and gained for go and no-go trials. At the end of each block, instructions emphasized either the need to withhold responses to the no-go or make timely responses to the go stimulus, depending upon whether participants achieved 50% successful inhibitions during the block.

Inhibition rates are presented in Table 2 and demonstrate the success of the algorithm in minimizing between-subject and between-session variance in inhibition rate. Because of the tracking algorithm, this index was blunted as a behavioral measure of inhibitory control. The median value of the RT cap for each condition was used as an alternative index: the lower the median cap, the harder the participant was trying to inhibit. To correct for between-subject and between-session differences in overall response speed, the floor RT was subtracted from the median cap for each condition, yielding the RT cap − floor differential. To assess the effect of motivational incentives on participant behavior, a rating scale was administered at the end of each testing session, asking the children to report which condition they “liked the most” and which condition made them “most careful.” The frequency with which each condition was chosen in each group and session was scored.

### Medication Effects

Participants in the ADHD group performed the go/no-go paradigm once on and once off their usual methylphenidate dose (order counterbalanced within the group). For the off medication session, they were required to stop taking their medication 36 hours beforehand. Group mean methylphenidate dose was 1.11 (SD = .42) mg/kg. Symptoms were assessed each time using the DuPaul *et al.* (38) ADHD-Rating Scale-IV; Table 1 shows a significant decrease in symptoms on medication compared with off medication.

### Procedure

Participants attended on two separate days, each consisting of an EEG session and a functional magnetic resonance imaging (fMRI) session (not reported here), with the order of testing counterbalanced and held constant across the 2 days. Attention-deficit/hyperactivity disorder participants were tested off and on medication; control subjects were never medicated but were tested twice to control for practice effects. Control (CTRL) and ADHD groups were pairwise matched on sociodemographic variables and on the order in which each pair completed EEG and fMRI testing. In between-group statistical analyses, the off-medication session of each ADHD participant was compared with the equivalent session of their matched control subject. Data from two participants in the ADHD group who did not complete the off-medication ERP session were excluded from analysis; the control participants with whom they were matched remained in the analysis to increase power.

### Electrophysiological Data Recording

Data were collected using a Biosemi Active II System (Biosemi, Amsterdam, Netherlands) with 128-channel montage of silver/silver chloride (Ag/AgCl) electrodes, sampled at 256 Hz. Additional electrodes were placed at the inner orbital ridge and the outer canthus of each eye to record eye movements and on each mastoid to record other artefacts. During data collection, voltage signals were referenced to an electrode placed to the left of electrode Cz.

### Electrophysiological Data Processing

Analysis was performed using Brain Vision Analyzer (BVA) 1.05 (Brain Products, Munich, Germany). After removal of noisy/flat channels, data were re-referenced to the average reference and filtered using .5 Hz high-pass and 30 Hz low-pass zero-phase shift Butterworth filters with slope of 24 dB per octave. Data were segmented into epochs, 2800 msec in length, with a prestimulus period of 650 msec. Large epochs were created at this stage to improve reliability of ocular correction, conducted using a regression method (39). Epochs with activity exceeding ±100 μV or less than 2.5 μV for more than 500 msec within an epoch were excluded. Baseline correction was performed using a 200 msec prestimulus reference period. Stimulus-locked epochs (−200 to 1000 msec peristimulus window) were averaged for the following trial types within each motivational condition: go (go trials with response within 100 to 900 msec of stimulus onset); no-go (all no-go trials). A number of participants in both groups (CTRL = 16; ADHD off medication = 20; ADHD on medication = 15) had too few SI trials (<20) for reliable ERP averaging. To avoid losing participants with insufficient trials, ERPs were measured in the no-go waveform, collapsed across SI and FI trials. A number of statistical procedures (Supplement 1) were performed to ensure this does not confound investigation of the experimental hypotheses.

Peak detection was performed using a semiautomated procedure in which peaks were detected automatically by the BVA software and checked by an expert blind to group status, according to the following criterion: N2 (maximal negative peak in a 220–400 msec time window) at Fz and Cz; P3 (mean amplitude in a 400–700 msec time window) at Cz and Pz. The early portion of the waveform often contained a negative shift; to provide a more reliable measure of N2 amplitude, the peak was calculated as the difference in amplitude between the N2 and the preceding positive peak. The P3 was measured as mean amplitude because in the majority of datasets, the activity in this time window occurred over a long time period and it was frequently difficult to identify one specific peak.

### Statistical Analysis

All analyses were conducted in two parts: first, effects of diagnosis were investigated by comparing the CTRL group with the ADHD group off medication. Second, effects of medication in the ADHD group were investigated by comparing the off and on medication sessions within subjects. In both analyses, motivation was a within-subjects factor with three levels (baseline, reward, and response cost). Analysis of ERP amplitudes included a within-subjects factor trial with two levels (go, no-go). Effect sizes (partial eta squared; η_p_^2^) are reported. Main effects and interactions significant at *p* < .05 (two-tailed) and trends (*p* < .1) were followed up with further analysis. Effects of motivation were investigated with planned Helmert contrasts comparing motivated conditions (reward and response cost) with baseline and reward with response cost.

Five participants (two ADHD off medication; two ADHD on medication; one ADHD both sessions) had fewer than 20 artifact-free no-go trials for ERP averaging and were excluded from analysis. Final sample sizes were 28 CTRL and 23 ADHD off medication for diagnostic analyses and 22 ADHD participants for medication analyses.

The data were checked for homogeneity of variance and normal distribution. Outliers (standard deviation greater than 2.5 from group mean) were excluded. The data were checked to ensure two female subjects in the sample (one ADHD, one CTRL) were not outliers; results remained robust to exclusion of these participants. All analyses described above were rerun with IQ and ODD diagnosis included as covariates. Neither were significant predictors in any analysis; the results are reported without the covariates included.

## Results

### Effects of Motivation on Visual Analogue Scales

Participants responded to the motivational manipulations within the paradigm, choosing the reward condition (CTRL: 91.1%; ADHD: 82%) above response cost (CTRL: 6.7%; ADHD: 7.7%) or baseline (CTRL: 2.2%; ADHD: 10.3%) as the condition they “liked the most.” Neither group (χ^2^ = 2.494, *p* > .1) nor session (χ^2^ = 3.209, *p* > .1) affected these choices. Both groups more frequently chose the response cost condition (CTRL: 81.8%; ADHD: 72.5%) than either reward (CTRL: 18.2%; ADHD: 17.5%) or baseline (CTRL: 0% ADHD: 10%) as the one that made them “most careful” with no significant group (χ^2^ = 4.641, *p* = .098) or session (χ^2^ = .082, *p* > .1) effects on these choices.

### Effects of Motivation on Performance

Data for the RT cap − floor differential are shown in Table 2. Statistical analysis conducted to investigate the effects of diagnosis and motivation on the RT differential revealed no effect of diagnosis [*F*(1,52) < 1, η_p_^2^ < .001] but a significant effect of motivation [*F*(2,104) = 10.972, *p* < .001, η_p_^2^ = .17] with smaller mean differential in the motivated conditions (reward and response cost) than baseline (*p* < .001). The diagnosis × motivation interaction was nonsignificant [*F*(2,104) < 1, η_p_^2^ = .009]. Analysis investigating the effects of medication and motivation on the differential revealed no effect of medication [*F*(1,25) < 1, η_p_^2^ = .03] but a significant effect of motivation [*F*(2,50) = 7.569, *p* = .001, η_p_^2^ = .23], with smaller differential in the motivated conditions than baseline (*p* = .006). The medication × motivation interaction was nonsignificant [*F*(2,50) = 2.920, *p* > .1, η_p_^2^ = .07].

### Effects of Diagnosis and Motivation on ERP Measures

**Figure 2:**
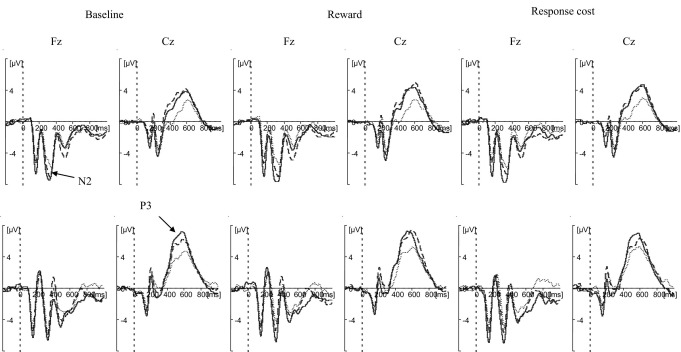
Stimulus-locked waveforms of CTRL group and ADHD group, off and on medication. Panels show waveforms for baseline condition (left), reward condition (center), and response cost condition (right). Within each panel, frontal electrode (Fz) is shown on the left and central electrode (Cz) is shown on the right. The top row shows go trials, and the bottom row shows no-go trials. Solid line represents the CTRL group; dotted line represents the ADHD group off medication; dashed line represents the ADHD group on medication. The waveform for the ADHD group off medication includes one participant excluded from the within-subjects medication comparison due to low trial numbers. ADHD, attention-deficit/hyperactivity disorder; CTRL, control.

Event-related potential data relevant to all statistical analyses are shown in Table 2. Event-related potential waveforms are shown in Figure 2.

#### N2

Analysis revealed significantly greater amplitude on no-go than go trials [*F*(1,49) = 22.065, *p* < .001, η_p_^2^ = .31] and a main effect of motivation [*F*(2,98) = 3.568, *p* = .032, η_p_^2^ = .06] with greater amplitude for reward than response cost (*p* = .005). Group differences were in the predicted direction (CTRL > ADHD) but did not reach significance [*F*(1,49) = 2.723, *p* = .1, η_p_^2^ = .05]. The diagnosis × motivation interaction [*F*(2,98) = 1.111, *p* > .1, η_p_^2^ = .022] and the diagnosis × motivation × trial interaction [*F*(2,98) < 1, η_p_^2^ = .011] were nonsignificant.

#### P3

There were significant main effects of diagnosis [*F*(1,49) = 5.164, *p* = .027, η_p_^2^ = .1], trial [*F*(1,49) = 33.364, *p* < .001, η_p_^2^ = .41], and motivation [*F*(2,98) = 5.118, *p* = .008, η_p_^2^ = .1] on P3 amplitude, with greater amplitude for CTRL than ADHD, for no-go than go trials, and for reward and response cost compared with baseline (*p* = .003). The diagnosis × motivation interaction [*F*(2,98) < 1, η_p_^2^ = .008] and the diagnosis × motivation × trial interaction [*F*(2,98) = 1.254, *p* > .1, η_p_^2^ = .025] were nonsignificant.

### Effects of Medication and Motivation on ERP Measures

#### N2

One extreme outlier was excluded. Amplitude was significantly greater on than off medication [*F*(1,20) = 5.844, *p* = .025, η_p_^2^ = .23] and for no-go than go trials [*F*(1,24) = 5.298, *p* = .03, η_p_^2^ = .18]. There was a main effect of motivation [*F*(2,40) = 4.752, *p* = .014, η_p_^2^ = .19] with greater amplitude in motivated conditions than baseline (*p* = .045) and in the reward condition compared with response cost (*p* = .036). The medication × motivation [*F*(2,48) = .621, *p* > .1, η_p_^2^ = .03] and medication × motivation × trial [*F*(2,48) = 2.314, *p* > .1, η_p_^2^ = .09] interactions were nonsignificant.

#### P3

Amplitude was significantly greater on than off medication [*F*(1,21) = 24.490, *p* < .001, η_p_^2^ = .54] and for no-go than go trials [*F*(1,21) = 17.691, *p* < .001, η_p_^2^ = .46]. There was a main effect of motivation [*F*(2,42) = 4.00, *p* = .02, η_p_^2^ = .16] with greater amplitude in both motivated conditions compared with baseline (*p* = .007) and no significant difference between reward and response cost (*p* > .1). The medication × motivation [*F*(2,50) = .52, *p* > .1, η_p_^2^ = .02] and medication × motivation × trial [*F*(2,50) = 1.374, *p* > .1, η_p_^2^ = .05] interactions were nonsignificant.

## Discussion

This study investigated the effects of motivational incentives and medication on electrophysiological indexes of response conflict and attention in children with ADHD. To ensure neural correlates were invoked to a similar degree in all children, a tracking algorithm minimized between- and within-subject variance in the ratio of failed to successfully inhibited no-go trials. Analysis of the self-report visual analogue scales confirmed that participants responded to the motivational incentives: participants in both groups and sessions chose the reward condition as the one they liked the most and the response cost condition as the one that made them most careful. The success of the tracking algorithm and the motivational incentives was critical to assessing effects of diagnosis, medication, and motivation on the ERPs.

### Effects of Diagnosis and Motivation

In support of previous research, the P3, an index of attention to a task-relevant stimulus (40) was significantly reduced in the ADHD group. N2 amplitude, an index of response inhibition (9) or conflict detection/resolution (10), was reduced, although this fell just short of statistical significance. Analysis revealed significant increases in N2 and P3 amplitudes in both groups in the motivated conditions compared with the baseline (low motivation) condition, indicating greater activation of processes underlying response conflict and attention to a task-relevant (salient) stimulus when the incentive to inhibit increased. Motivational incentives enhanced amplitudes of the ADHD group, bringing them closer to the baseline amplitudes of the control group. There was no interaction between diagnosis and motivation. In previous behavioral studies (17–23), the absence of interaction has been interpreted as evidence of independence of inhibitory and motivational deficits in ADHD, supporting the dual pathway model (24). The present findings clearly show, however, that motivational incentives were effective in enhancing electrophysiological markers of inhibitory control in ADHD, although the effects were not differentially greater than in control subjects. This suggests first that, in support of previous studies, the ADHD children were neither hypersensitive nor hyposensitive to these immediate, consistent incentives (12) and second that, to achieve the same level as control children in both the baseline and motivated conditions, children with ADHD may require incentives that differ quantitatively or qualitatively from those that produce effects in typically developing children. It will therefore be important in future research to investigate other factors that influence the effectiveness of incentives on inhibitory control in ADHD, such as reward magnitude, reinforcement history, and reinforcement scheduling (11,12). This may help clarify the nature of relationships between inhibitory and motivational deficits in this population. Of note, the diagnosis effect for the N2 did not quite reach statistical significance, possibly providing insufficient scope for measuring relationships between this index and motivation.

### Effects of Medication and Motivation

Within-subjects analysis revealed significant effects of medication on N2 and P3 amplitudes, supporting the relatively limited research in this area (27–31) and identifying a possible locus for the effects of methylphenidate on symptom severity. Interactions were statistically nonsignificant, suggesting that medication and motivation have additive, rather than synergistic, effects on these neural correlates. With regard to the MTA study, which reported that stimulant medication was effective at lower doses when combined with behavioral therapy (32), our findings suggest these interventions may act independently. Follow-up in the MTA revealed that behavioral treatment alone was less effective than medication alone, a finding that is consistent with the larger effect sizes for medication than motivational incentives in the present study. Of course, the motivational incentives employed here were not designed to replicate behavioral modification programs used in clinical practice, but the results suggest that both operant conditioning (reward and response cost) and stimulant medication impact these particular electrophysiological indexes of response conflict and allocation of attentional resources. It will also be important to determine whether the effects identified in the present study are generalizable to other response inhibition paradigms and to children with less stringently defined ADHD, including those with the inattentive subtype.

### Limitations

Although the sample sizes were large compared with many other ERP studies in this field (e.g., [5,7,17]), statistical power may have been insufficient to detect critical interactions. However, several methodological steps were taken to maximize power: careful matching of samples on age, gender, and SES; conducting analysis on the equivalent session for each matched CTRL-ADHD pair; tracking algorithm to reduce between-subjects variance; and careful preprocessing of EEG data. Power calculations revealed 79% power to detect an effect size of .4 and 84% power to detect an effect of .3 for the diagnosis by motivation and medication by motivation interactions, respectively. Nonsignificant interactions are therefore likely to be small. Low numbers of successfully inhibited no-go trials meant it was not possible to investigate neural correlates specific to successful inhibition. Previous studies have identified differences in the morphology of the waveform on FI and SI trials, particularly with regard to the amplitude and latency of the N2 and P3 (9,10). A number of additional analyses (Supplement 1) were conducted to ensure that collapsing across FI and SI trials does not confound interpretation of the effects of diagnosis, medication, or motivation presented here.

### Conclusions

This is the first study to report effects of motivation on ERP correlates of response conflict and attention in children with ADHD. Stimulant medication also increased N2 and P3 amplitude. Methylphenidate treatment and motivational incentives had independent rather than synergistic effects on these ERP markers, with relatively greater effects of medication than motivation. Future research should investigate under controlled conditions whether the additive effects reported here for motivational incentives and stimulant medication in ADHD is replicated for behavioral and symptomatic outcomes.

## Figures and Tables

**Table 1:** Clinical and Demographic Characteristics of CTRL and ADHD Groups

	CTRL (*n* = 28)		ADHD (*n* = 28)		Comparison	
	Mean	SD	Mean	SD	*t* (*df* = 54)	*p*
Age	12.54	1.81	12.51	1.75	.07^a^	.946
IQ	104.93	14.31	90.86	11.71	4.027	<.001
SDQ						
Hyperactive	1.68	1.70	9.00	1.66	16.32^a^	<.001
Conners						
DSM-Hyperactive	43.64	3.27	84.96	7.23	25.37^a^	<.001
DSM-Inattentive	43.65	3.22	73.50	7.96	18.36^a^	<.001
DSM-Total	44.25	3.32	81.61	7.69	23.62^a^	<.001
ADHD-RS^c^						
Off medications	—	—	70.08	4.24	2.92^b^	.004
On medications	—	—	64.31	10.51		
Order of Testing^d^						
Off medications first	13		13			
On medications first	15		15			

ADHD, attention-deficit/hyperactivity disorder; ADHD-RS, ADHD-Rating Scale-IV; Conners, Conners Rating Scale-Revised (Long Form) ADHD index; CTRL, control group; DSM, Diagnostic and Statistical Manual of Mental Health Disorders; IQ, intelligence quotient; SDQ, Strengths and Difficulties Questionnaire.

a Independent-samples *t* test comparing control group (CTRL) and ADHD group.

b Paired-samples *t* test comparing off and on medication scores in the ADHD group; this analysis was conducted on 26 participants who completed both the off and on medication sessions.

c Scores on the DuPaul ADHD Rating Scale were converted to *t* scores using published population percentiles (38).

d Order of testing was counterbalanced and randomized within the ADHD group. Participants in the control group were never medicated but were assigned to the same order of testing as the ADHD participant with whom they were pairwise-matched.

**Table 2:** Descriptive Data for Behavioral Measures and ERP Amplitudes in CTRL and ADHD Groups

		Diagnosis				Medication (*n* = 22)			
		CTRL (*n* = 28)		ADHD (*n* = 23)		ADHD Off Medication		ADHD On Medication	
		Mean	SD	Mean	SD	Mean	SD	Mean	SD
RT Cap − Floor Differential (msec)	Baseline	328.92	182.05	337.94	171.91	339.34	166.28	280.70	213.96
	Reward	264.18	172.68	237.46	165.45	240.48	159.96	208.54	184.56
	Response Cost	235.17	175.18	244.67	165.72	254.11	169.32	247.75	208.71
Inhibition Rate	Baseline	39.57	15.53	39.08	15.23	39.08	15.23	44.54	18.34
	Reward	46.50	13.14	47.23	15.60	47.23	15.60	51.31	17.05
	Response Cost	44.86	13.08	45.23	13.57	45.23	13.57	47.08	15.83
N2 Go Trials (μV)	Baseline	−9.37	4.15	−7.92	3.83	−7.74	3.74	−9.65	3.56
	Reward	−9.30	4.22	−8.82	3.71	−8.83	3.84	−9.79	3.69
	Response Cost	−9.02	3.96	−8.17	3.49	−7.87	3.36	−9.48	3.88
N2 No-Go Trials (μV)	Baseline	−12.16	4.64	−9.69	4.56	−8.83	3.36	−9.47	3.81
	Reward	−12.47	5.03	−10.25	3.91	−9.57	3.32	−10.47	3.59
	Response Cost	−12.13	4.75	−9.36	4.14	−8.87	3.88	−10.51	3.89
P3 Go Trials (μV)	Baseline	3.56	2.44	2.10	2.73	2.15	2.78	4.15	2.86
	Reward	4.03	2.56	2.24	2.57	2.24	2.63	4.73	2.98
	Response Cost	4.17	2.60	2.53	2.85	2.52	2.92	4.48	2.67
P3 No-Go Trials (μV)	Baseline	6.10	3.49	3.86	3.50	3.89	3.58	6.04	3.66
	Reward	6.37	3.58	4.46	3.95	4.46	4.04	6.74	3.41
	Response Cost	6.14	3.72	4.54	3.70	4.42	3.74	6.09	3.67

Values for inhibition rate must be interpreted in the context of the tracking algorithm employed within the paradigm that minimized between-subject and between-session differences in the ratio of failed to successfully inhibited no-go trials. ADHD, attention-deficit/hyperactivity disorder; CTRL, control group; ERP, event-related potential; RT, reaction time.
