# Supplementary material for: Effects of Motivation and Medication on Electrophysiological Markers of Response Inhibition in Children with Attention-Deficit/Hyperactivity Disorder
Source: Biol Psychiatry. 2010 Apr 1;67(7):624–31. doi: 10.1016/j.biopsych.2009.09.029 (PMC2845810; doi:10.1016/j.biopsych.2009.09.029)
Supplement: Supplement 1 [file mmc1.pdf]

Supplemental Information

Statistical analyses to investigate effects of trial type

A number of participants in both groups (CTRL=16; ADHD off medication=20; ADHD on medication=15) had too few SI trials (<20) for reliable ERP averaging. To avoid losing participants with insufficient SI trials (and therefore compromising the generalizability of our findings to children with ADHD with poor inhibition) ERPs were measured in the no-go waveform, collapsed across SI and FI trials. The N2 and P3 measured in the present study are therefore electrophysiological indices of processes common to all no-go trials, regardless of inhibitory failure or success. The typical approach to analyzing ERPs measured in a go/no-go task is to compare correct go trials with correct (successfully inhibited) no-go trials on the basis that the latter provide a measure of the neural processes related to successful response inhibition. To ensure that in this study the decision to collapse across trial types does not confound investigation of the effects of Diagnosis, Medication and Motivation on the N2 and P3, the following statistical checks were under-taken.

Firstly, analysis conducted in a sub-sample of participants (CTRL=14; ADHD=9) with sufficient numbers of trials (>20) for separate ERP averaging of SI and FI trials revealed no significant differences between these trial types either for N2 (mean FI = -11.76μV; mean SI = -11.56μV) [F (1, 15) <1] or P3 (mean FI = 4.55μV; mean SI = 3.78μV) [F (1, 15) = 2.72, p>.1]. Although the trial type effect did not reach significance for either ERP, mean P3 amplitude is slightly greater on FI than SI trials. However, effects of Diagnosis and Medication on P3 amplitude are unlikely to be confounded by this small amplitude difference between trial types: there were no significant differences between groups [t (1, 49) = .12, p>.1] or medication sessions [t (1, 21) = -1.40, p>0.1] in the proportion of FI and SI trials contributing to the no-go waveform. The trial type difference is also unlikely to confound

interpretation of effects of motivation on P3 amplitude: inhibition rate was significantly greater in the motivated conditions compared with baseline across groups [F (2,104) = 13.842, p<.001] meaning that the proportion of FI to SI trials is lower in the motivated conditions. Given that P3 amplitude is larger on FI than SI trials, any possible confound of trial type effects will therefore reduce, rather than inflate, the effects of motivation on P3 amplitude.

Secondly, correlations between the ratio of FI to SI trials included in the no-go waveform and N2 and P3 amplitudes and N2 latencies on no-go trials were statistically non-significant in all motivational conditions (all r< 0.14) indicating that these parameters of the no-go waveform do not vary with the proportion of failed to successful no-go trials included in the waveform.

Finally, the mean of the FI and SI peak amplitudes was computed for each motivational condition in each dataset. This index gives equal weighting to amplitudes measured on FI and SI trials, ensuring that electrophysiological processes on FI and SI trials are equally represented. Analyses were repeated using this index in place of the peak amplitudes measured on no-go trials. All results reported in the manuscript remained robust to the use of this index supporting the decision to measure electrophysiological processes common to all no-go trials.

These analyses indicate that in this particular paradigm, it is appropriate to investigate effects of Diagnosis, Motivation and Medication on electrophysiological processes common to all no-go trials, for both the N2 and P3.
